# Disruption of reducing pathways is not essential for efficient disulfide bond formation in the cytoplasm of *E. coli*

**DOI:** 10.1186/1475-2859-9-67

**Published:** 2010-09-13

**Authors:** Feras Hatahet, Van Dat Nguyen, Kirsi EH Salo, Lloyd W Ruddock

**Affiliations:** 1 Department of Biochemistry, Linnanmaa Campus, University of Oulu, 90570 Oulu, Finland

## Abstract

**Background:**

The formation of native disulfide bonds is a complex and essential post-translational modification for many proteins. The large scale production of these proteins can be difficult and depends on targeting the protein to a compartment in which disulfide bond formation naturally occurs, usually the endoplasmic reticulum of eukaryotes or the periplasm of prokaryotes. It is currently thought to be impossible to produce large amounts of disulfide bond containing protein in the cytoplasm of wild-type bacteria such as *E. coli *due to the presence of multiple pathways for their reduction.

**Results:**

Here we show that the introduction of Erv1p, a sulfhydryl oxidase and FAD-dependent catalyst of disulfide bond formation found in the inter membrane space of mitochondria, allows the efficient formation of native disulfide bonds in heterologously expressed proteins in the cytoplasm of *E. coli *even without the disruption of genes involved in disulfide bond reduction, for example *trxB *and/or *gor*. Indeed yields of active disulfide bonded proteins were higher in BL21 (DE3) pLysSRARE, an *E. coli *strain with the reducing pathways intact, than in the commercial Δ*gor *Δ*trxB *strain rosetta-gami upon co-expression of Erv1p.

**Conclusions:**

Our results refute the current paradigm in the field that disruption of at least one of the reducing pathways is essential for the efficient production of disulfide bond containing proteins in the cytoplasm of *E. coli *and open up new possibilities for the use of *E. coli *as a microbial cell factory.

## Background

Disulfide bond formation is one of the most common types of protein post-translational modification, with disulfide bonds being found in most outer membrane or secreted proteins. The formation of native disulfide bonds is not trivial and complex pathways have evolved in the three cellular compartments in which catalyzed disulfide bond formation commonly occurs, the endoplasmic reticulum (ER) of eukaryotes [1], the inter-membrane space of mitochondria [2] and the periplasm of prokaryotes [3]. These pathways include components that catalyze the formation of disulfide bonds and others that catalyze the subsequent rearrangement or isomerization of incorrect or non-native disulfide bonds. Despite the presence of these catalyzed pathways for forming protein disulfides native disulfide bond formation is often the rate-limiting step in protein folding *in vitro *and *in vivo*.

**Figure 1:**
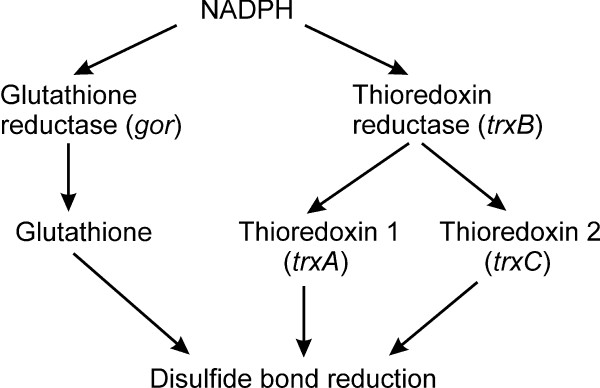
**Dual pathways for disulfide bond reduction in the cytoplasm of *E. coli***. Dual pathways for disulfide bond reduction in the cytoplasm of *E. coli*. On the knockout of both pathways the reduction of disulfide bonds is inhibited, but there is no active system to catalyze their formation.

In contrast to the compartments in which catalyzed disulfide bond formation occurs, the environment of the cytoplasm of most prokaryotes has evolved not only lacking components that catalyze formation of disulfide bonds, but also having active systems that result in the reduction of protein disulfide bonds (Figure 1). Due to the presence of these pathways the production of proteins that contain disulfide bonds is thought to be impossible in the cytoplasm of most wild-type prokaryotes such as *E. coli *[4]. When such proteins are expressed they are unable to attain their native conformation and commonly form insoluble aggregates known as inclusion bodies. While such inclusion bodies can be purified and refolded, it would be useful to have a system for large scale production of disulfide bond containing proteins in the cytoplasm of *E. coli*.

To circumvent the problem associated with the production of disulfide bond formation in the cytoplasm of *E. coli *a variety of modified strains have been produced [5-9]. These strains, which evolved from seminal studies on *E. coli *physiology, have a total or partial disruption of one or both of the pathways involved in ensuring that the cytoplasm is reducing. Strains in which both pathways are disrupted show a significant growth defect connected with the reducing pathways being required for other cellular processes e.g. the function of ribonucleotide reductase, unless the media is supplemented with a reducing agent [6]. However, this requirement in rich media can be obviated by spontaneous mutations in *aphC *[9-11]. The disruption of these two pathways through a knockout of the two NADPH dependent reductases *trxB *and *gor *combined with a mutation in *aphC *allows for a significant increase in the production of activity of even a complex disulfide bonded protein such as a truncated variant of tissue plasminogen activator [vtPA; 8,12]. The addition of DsbC, a periplasmic disulfide isomerase [13], increased the yields of active vtPA produced a further 20-fold [8]. Such Δ*gor *Δ*trxB *strains with mutations in *aphC *are available commercially, for example origami or rosetta-gami (Novagen) or with DsbC co-expression as the SHuffle system (New England Biolabs). However, the yields of many disulfide bonded proteins from these systems are below that required for commercial production or even for the production of proteins for academic studies.

While Δ*gor *Δ*trxB *strains lack the reducing pathways and hence allow thioredoxins to transfer oxidizing equivalents from other metabolic pathways to folding proteins [7], there is no dedicated source or catalyst for disulfide bond formation. This is in contrast to compartments in which disulfide bond formation occurs. These all naturally have catalysts of *de novo *disulfide bond formation, such as the sulfhydryl oxidases Ero1, found in the ER [14], or Erv1p, found in the mitochondrial inter-membrane space of *S. cerevisiae *[15] which can catalyze the reaction:

$$\text{Dithiol}+{\text{O}}_{2}\overset{}{\to }\text{Disulfide}+{\text{H}}_{2}{\text{O}}_{2}$$

or the transmembrane protein DsbB required for disulfide bond formation in the periplasm [16] which transfers electrons to menaquinone or ubiquinone. While all of the catalysts of disulfide bond formation are thought to act via an intermediary protein, protein disulfide isomerase (PDI) for Ero1 [17], Mia40 for Erv1p [18] and DsbA for DsbB [16], rather than directly on non-native protein substrates, data exists in the literature that Erv1p may be able to function independently of Mia40 [for example 19,20]. In addition, we have observed that Erv1p is able to efficiently oxidatively refold reduced denatured bovine pancreatic trypsin inhibitor to a two disulfide state in the absence of any other factor such as Mia40, glutathione, thioredoxin or PDI (unpublished observations). Hence Erv1p may be a suitable single protein system to catalyse *de novo *disulfide bond formation.

To date all reported systems for making disulfide bond containing proteins in the cytoplasm of *E. coli *are based on the disruption of one, or more usually both, of the naturally occurring reduction pathways. Here we show that the introduction of the sulfhydryl oxidase Erv1p, an enzyme which can use molecular oxygen to catalyze the oxidation of a dithiol to a disulfide in an FAD-dependent manner, into the cytoplasm of wild-type *E. coli *results in as high or higher levels of production of active proteins than a Δ*gor *Δ*trxB *strain. Hence our results refute the current paradigm in the field that disruption of at least one of the reducing pathways is essential for the efficient production of disulfide bond containing proteins and opens up new possibilities for their production.

## Results

### Alkaline phosphatase production

**Figure 2:**
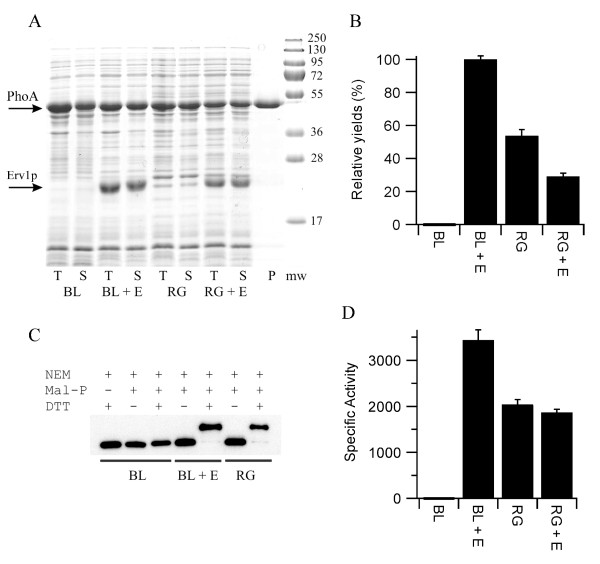
**Production of PhoA in the cytoplasm of *E. coli***. A) SDS-PAGE analysis of the production of PhoA produced in LB media at 30°C. T = total *E. coli *lysate, S = *E. coli *lysate soluble fraction, P = purified protein from BL21 with co-expression of Erv1p from the NEM-treated lysate and is representative of the quality obtained from all of the samples. Mw = molecular weight markers. BL = BL21 (DE3) pLysSRARE; RG = rosetta-gami; + E = co-expression of *S. cerevisiae *Erv1p from a polycistronic vector. The positions of PhoA (upper) and Erv1p (lower) are marked with arrows. B) Relative yields of active PhoA normalized to the system producing the most active protein and shown as percentage mean ± s.d. (n = 4). C) Representative blot from a shift-assay based on alkylation of free thiol groups to examine the disulfide bond status of the PhoA produced. Note that the samples are treated with the thiol-blocking agent N-ethylmaleimide (NEM) before reduction and maleimide based addition of polyethyleneglycol. Hence an increase in apparent molecular weight is consistent with the presence of one or more disulfide bonds in the original sample. The greater the number of disulfide bonds the greater the mass shift. PhoA produced in both the Δ*gor *Δ*trxB *background and in the wild-type background plus co-expression of Erv1p show a homogeneous disulfide bonded protein being produced, however this assay does not determine whether these disulfide bonds are native or not. D) Specific activity (μmole of product formed per minute per mg of protein) of purified PhoA shown as mean ± s.d. (n = 3).

Our initial screen for whether co-expression of the sulfhydryl oxidase Erv1p could increase the yield of disulfide bond containing proteins was based on *E. coli *alkaline phosphatase (PhoA), a protein which is widely used to examine disulfide bond formation *in vivo*. PhoA is a hydrolase which naturally folds in the periplasm and contains two sequential disulfide bonds whose formation is essential for activity [21]. While the activity of PhoA has an essential requirement for disulfide bond formation, when expressed in a reducing environment such as the cytoplasm of *E. coli *the protein does not form inclusion bodies. Hence, expression of the mature form of PhoA i.e. the protein lacking the periplasmic signal sequence, in the cytoplasm of *E. coli *resulted in high levels of soluble protein being produced (Figure 2A), but in negligible yields of active protein (Figure 2B and Table 1), consistent with the reducing environment of the cytoplasm. In contrast, co-expression of Erv1p with PhoA from a polycistronic vector resulted in high yields of active PhoA (Figure 2B and Table 1). Exact quantification of the fold increase is difficult, given the absence of recombinant PhoA activity when Erv1p is not co-expressed, but it is at least 1000-fold. Furthermore, the yield of active PhoA upon co-expression of Erv1p in the *E. coli *strain BL21(DE3) pLysSRARE, which has the reducing pathways in place, was circa two-fold higher than that produced in the equivalent commercial Δ*gor *Δ*trxB *strain rosetta-gami (Figure 2B) despite similar amounts of protein being produced (Figure 2A). Analysis of the PhoA produced in these strains via gel-shifts after mal-PEG treatment [22] implied that effectively all of the PhoA produced in the cytoplasm of the *E. coli *strain BL21 upon Erv1p co-expression contained disulfide bonds (Figure 2C), despite the protein being produced in a strain with both disulfide reducing pathways intact.

**Table 1:** Production of PhoA in the cytoplasm of *E. coli*

Sample	Δ Absorbance (mAU/min)	Final OD of culture	Relative yield (%)
PhoA in BL21 (DE3)	-0.068 ± 0.053	2.67 ± 0.20	-0.3 ± 0.3

PhoA + Erv1p in BL21 (DE3)	27.6 ± 0.6	1.73 ± 0.01	100 ± 2.1

PhoA in rosetta-gami	16.2 ± 0.6	1.59 ± 0.05	53.9 ± 3.6

PhoA + Erv1p in rosetta-gami	11.0 ± 0.8	1.27 ± 0.03	29.3 ± 1.8

Data for the production of active *E. coli *alkaline phosphatase (PhoA) grown in LB media in shake flasks at 30°C, where Δ absorbance is the rate of change in absorbance per minute at 410 nm upon assaying the lysate (n = 4). Note that both the BL21 strain and the rosetta-gami (Δ*gor *Δ*trxB*) strain contain pLysSRARE. As can be seen from the data the growth rate of both the BL21(DE3) pLysSRARE and rosetta-gami strains decreases upon co-expression of Erv1p resulting in a lower final OD600 of the culture after 4 hours of induction. Despite this the relative yield of active protein is much higher upon co-expression.

To further characterize the system, PhoA from all four expression conditions was purified by immobilised metal affinity chromatography. This one step purification resulted in highly purified protein (Figure 2A). Reduced PhoA is prone to air oxidation which results in the formation of disulfide bonds and a gain in activity. To ensure the specific activity measurements of PhoA were not influenced by this, while allowing analysis of the free thiol content of the purified protein, part of the *E. coli *lysate was treated with N-ethyl maleimide (NEM) to block free thiol groups and prevent *ex vivo *disulfide bond formation, while another part was not. Parallel purifications of the two samples from each of the four expression conditions were performed. Despite neither the expression conditions nor the purification procedure being optimised the equivalent of 16 mg/L of PhoA was obtained from the *E. coli *strain BL21 with co-expression of Erv1p. Activity measurements on the PhoA protein from the NEM-blocked samples showed that the specific activity of the PhoA from the *E. coli *strain BL21 was circa 1000-fold greater when Erv1p was co-expressed and that this material was circa 1.7-fold more active that the PhoA purified from the rosetta-gami strain (Figure 2D). A similar pattern was observed for the PhoA purified from the non-NEM treated lysate except that the specific activity of the PhoA from the BL21 strain was circa 3-fold greater than that from the non-treated lysate, consistent with *ex vivo *oxidation. An Elman's assay of the non-NEM treated purified samples, under denaturing conditions, revealed that the PhoA purified from the BL21 strain had circa 3.1 free thiol groups per protein, while there was no detectable free thiol groups on the protein purified from the same strain with co-expression of Erv1p. Electrospray mass spectrometric analysis of non-treated samples showed that the major component for all four proteins were of the expected mass of PhoA (48285 Da; mass accuracy 0.01%), but additional minor peaks were also observed. For all four samples an additional peak of +80Da was observed in the mass spectra, consistent with the addition of a phosphate group. The relative intensity of this peak was around 25% except for the PhoA purified from the BL21 strain in the absence of Erv1p co-expression where it was around 10%. For both of the PhoA samples purified from the BL21 strains an additional peak consistent with gluconoylation of the N-terminal hexa-histdine tag [23] was also observed, with this post-translational modification also being found on around 25% of the total protein. It is unclear to what extent this additional post-translational modification, that we commonly observe for N-terminal hexa-histidine tagged proteins expressed in BL21, affects the activity of PhoA. Both SDS-PAGE and mass spectrometry revealed that the PhoA purified from the BL21 (DE3) strain in the absence of Erv1p co-expression became increasingly degraded upon storage, something not observed for the other samples over the same time scale. This is consistent with the lack of disulfide bonds in this protein.

### Phytase production

**Figure 3:**
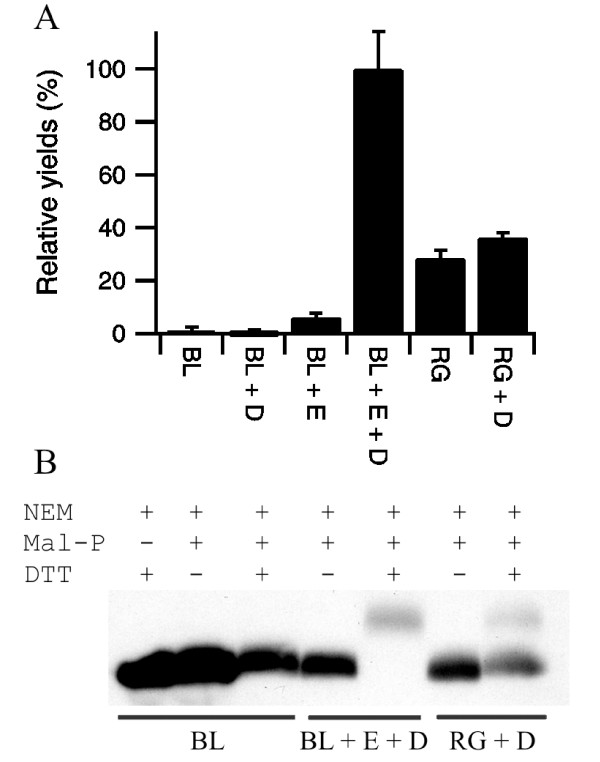
**Production of AppA in the cytoplasm of *E. coli***. A) Relative yields of active AppA produced in LB media at 30°C, normalized to the system producing the most active protein and shown as percentage mean ± s.d. (n = 4). BL = BL21 (DE3) pLysSRARE; RG = rosetta-gami; + = co-expression from a polycistronic vector where D = mature *E. coli *DsbC, E = *S. cerevisiae *Erv1p. B) Representative blot from a shift-assay based on alkylation of free thiol groups to examine the disulfide bond status of the AppA produced. While AppA produced upon co-expression of Erv1p in a wild-type background shows a homogeneous disulfide bonded protein being produced, the protein produced in the Δ*gor *Δ*trxB *background shows heterogeneity and a lower degree of disulfide bond formation. Note that the molecular weight of the mal-PEG is not homogenous and hence modified proteins, especially those with multiple mal-PEG added, appear as more defuse bands.

PhoA is regarded as a very simple model substrate protein since it folds, though is not active, in the absence of disulfide bond formation and since disulfide bond formation in this protein is facile. As a more robust test of our system we switched to examining Phytase (AppA) production. AppA is an *E. coli *periplasmic protein with similar activity to PhoA except that it has optimal activity under acidic pH conditions. It contains four disulfide bonds one of which is non-sequential; as such it is used as a model protein for native disulfide bond formation which requires isomerization [24]. The expression of endogenous AppA is highly regulated and exogenous AppA is not highly expressed. Hence when the mature form of AppA is expressed in the cytoplasm of the *E. coli *strains BL21(DE3) pLysSRARE or rosetta-gami with or without co-expression of the sulfhydryl oxidase Erv1p and/or the disulfide isomerase DsbC from polycistronic vectors, the yield of AppA is so low that it cannot be detected by coomassie stained SDS-PAGE (data not shown), while DsbC and Erv1p showed good expression (as per Erv1p in Figure 2A). Detection of AppA is possible by western blot (see Figure 3B). Activity measurements from these lysates showed that active protein was expressed under certain conditions. When the mature form of AppA is expressed in the cytoplasm of the *E. coli *strains BL21(DE3) pLysSRARE with or without co-expression of the disulfide isomerase DsbC from a polycistronic vector negligible yields of active protein were obtained (Figure 3A and Table 2). However, co-expression of Erv1p from a polycistronic vector significantly increased the AppA activity produced, with the highest yield being obtained in BL21(DE3) pLysSRARE background upon co-expression of Erv1p and DsbC (Figure 3A and Table 2) i.e. once both a catalyst of disulfide bond formation (Erv1p) and of isomerization (DsbC) were expressed. Again absolute quantification of the increase in yield of active protein is difficult due to the negligible amounts of active protein produced in BL21(DE3) pLysSRARE when Erv1p is not co-expressed. However, the yields of active AppA produced with co-expression of Erv1p and DsbC in BL21(DE3) pLysSRARE are circa three-fold higher than those produced in the Δ*gor *Δ*trxB *strain rosetta-gami with co-expression of DsbC (Figure 3A). Furthermore, analysis of the AppA produced via gel-shift assay showed that the AppA produced by co-expression of Erv1p and DsbC in a wild-type *E. coli *background was comprised only of disulfide containing protein that migrated at the same position in the gel-shift assay, while that produced in the Δ*gor *Δ*trxB *strain with or without co-expression of DsbC resulted in a mixture of species with different numbers of disulfide bonds (Figure 3B) implying incomplete oxidation.

**Table 2:** Production of AppA in the cytoplasm of *E. coli*

Sample	Δ Absorbance	Final OD of culture	Relative yield (%)
AppA in BL21 (DE3)	0.003 ± 0.004	2.60 ± 0.18	1.3 ± 1.3

AppA + DsbC in BL21 (DE3)	-0.003 ± 0.004	2.02 ± 0.07	-0.7 ± 1.2

AppA + Erv1p in BL21 (DE3)	0.039 ± 0.009	1.10 ± 0.02	6.2 ± 1.4

AppA + Erv1p + DsbC in BL21 (DE3)	0.420 ± 0.062	1.65 ± 0.01	100 ± 14.2

AppA in rosetta-gami	0.105 ± 0.020	1.91 ± 0.14	28.6 ± 3.0

AppA + DsbC in rosetta-gami	0.123 ± 0.007	2.03 ± 0.01	36.2 ± 1.9

Data for the production of active *E. coli *phytase (AppA) grown in LB media at 30°C, where Δ absorbance is the change in absorbance over 20 minutes at 410 nm upon assaying the lysate (n = 4). Note that both the BL21 (DE3) strain and the rosetta-gami (Δ*gor *Δ*trxB*) strain contain pLysSRARE. As can be seen from the data the growth rate of both the BL21(DE3) pLysSRARE and rosetta-gami strains decreases upon co-expression of Erv1p resulting in a lower final OD600 of the culture after 4 hours of induction. Despite this the relative yield of active protein is much higher upon co-expression.

Purification of AppA from these strains is complicated by the very low expression levels. Immobilised metal affinity chromatography from a standard culture did not generate sufficient protein for the concentration of the purified AppA to be determined accurately. However, an enzyme activity assay performed immediately after purification revealed that very significant AppA activity was recovered from the BL21 (DE3) pLysSRARE strain with co-expression of Erv1p and DsbC. Relative quantification of the proteins purified using tryptophan intrinsic fluorescence showed that the protein purified from BL21(DE3) pLysSRARE had less than 1% of the relative activity of that obtained upon co-expression of Erv1p and DsbC, while that from rosetta-gami with co-expression of DsbC was 31%. These results on the purified proteins mirror the results from the *E. coli *lysates shown in Figure 3A. More than 99% of the AppA activity was lost from all of the purified samples upon a single freeze-thaw cycle in the purification elution buffer.

## Discussion

The current systems for making disulfide bonded proteins in the cytoplasm of *E. coli *are based on the disruption of one, or more usually both, of the reducing pathways normally present in this compartment [5-8], with a concomitant mutation in *aphC *[9-11] which restores growth rates in rich media. While these strains can produce much higher levels of disulfide bonded proteins than the wild-type strains the yields and/or quality are often not optimal even when a disulfide isomerase is co-expressed. While thioredoxin is reported to be involved in disulfide bond formation in Δ*trxB *strains [7], it is a catalyst of thiol-disulfide exchange and not a catalyst of *de novo *disulfide bond formation. Hence thioredoxin becomes oxidised in this system by reducing substrates such as ribonucleotide reductase and transfers the disulfide to folding proteins such as PhoA, such that disulfide bond formation in these strains can potentially be seen as being a "by-product" of other metabolic processes. In contrast, compartments that naturally produce disulfide bonded proteins have a catalytic system for making disulfide bonds for example a sulfhydryl oxidase which can use molecular oxygen to catalyze the oxidation of a dithiol to a disulfide. The cytoplasm of *E. coli *lacks such a catalyst. Hence, we thought that the introduction of such a system might significantly increase the yields of active disulphide-bonded protein produced. This turns out to be the case. Indeed the production of disulfide bonded proteins in the cytoplasm of *E. coli *does not require disruption of genes involved in either of the reducing pathways. Instead for PhoA and AppA the addition of a catalytic system for the formation of disulfide bonds i.e. co-expression of the sulfhydryl oxidase Erv1p, while not significantly altering the amount of recombinant protein produced, significantly increases the yields of active protein produced. Furthermore, it is more effective than the removal of the reducing pathways for the production of active PhoA and AppA. The expression conditions and purification reported here have not been optimized, but yields of 16 mg/L of culture of purified disulfide bonded protein were obtained from the cytoplasm of *E. coli *grown in shake flasks. Furthermore, our preliminary results indicate that such yields are possible for a range of other proteins of academic and commercial interest with multiple disulfide bonds (data not shown).

The ability of co-expression of Erv1p (plus a disulfide isomerase where needed) to enable the production of natively folded disulfide bond containing proteins in the cytoplasm of wild-type *E. coli *may seem contradictory with what is known about the environment of this cellular compartment. However, it is compatible with what is known about disulfide bond formation in other compartments. Neither the ER nor the inter-membrane space of mitochondria can be accurately described as an oxidizing environment. While the redox potential of the ER is more oxidizing than that of the cytoplasm it is still a compartment in which disulfide bond reduction and isomerization must occur [1] i.e. both oxidative and reducing pathways must co-exist in the same compartment. However, native disulfide bonds in folded proteins are often buried and hence inaccessible to reduction by either glutathione or members of the thioredoxin superfamily. Hence in our system there is probably a kinetic competition between oxidation/folding and reduction with the native disulfide bonded state being stable once formed even in the background of wild-type cytoplasm with both the thioredoxin and glutathione based reducing pathways intact.

The ability of Erv1p to act in the absence of Mia40 may also seem contradictory with what is known about its mechanism of action in the inter-membrane space of mitochondria. It is possible that Erv1p may be acting in our system via an intermediary molecule, such as one of the thioredoxins or glutaredoxins or via a low molecular weight species. However, there is published data that Erv1p is able to act without Mia40 [for example 19,20] and we have preliminary *in vitro *data that Erv1p is able to efficiently oxidise dithiols to disulfides in reduced unfolded proteins in the absence of any factors except molecular oxygen. The elucidation of the exact mechanisms by which Erv1p is acting in the cytoplasm of *E. coli *is not trivial, but this and a more detailed understanding of the other processes that are occurring may help in the optimization of this system for the production of recombinant disulfide bond containing proteins.

## Conclusions

By mimicking natural systems, which have both reducing and oxidizing pathways in place in compartments where oxidative folding occurs, we are able to generate more efficient production of disulfide bond containing proteins in the cytoplasm while at the same time avoiding the problems associated with the disruption of the reducing pathways e.g. the requirement of the reducing pathways for other cellular processes such as the function of ribonucleotide reductase. Furthermore, our results refute the current paradigm in the field that disruption of at least one of the reducing pathways is essential for the efficient production of disulfide bond containing proteins in the cytoplasm of *E. coli *and open up new possibilities for the use of *E. coli *as a microbial cell factory.

## Methods

### Vector construction

Expression vectors (Table 3) were made by standard molecular biology techniques.

**Table 3:** Vectors reported in this study

Plasmid	Basis	Protein being produced	Co-expression
pFH198	pET23	Mature *E. coli *DsbC (Asp21-Lys236)	-

pVD157	pET23	*S. cerevisiae *Erv1p (Met 1-Glu189)	-

pVD158	pET23	*S. cerevisiae *Erv1p (Met 1-Glu189)	DsbC

pVD80	pET23	MH_6_M-mature *E. coli *PhoA (Arg22-Lys471)	-

pVD82	pET23	MH_6_M-mature *E. coli *PhoA (Arg22-Lys471)	Erv1p

pVD96	pET23	MH_6_M-mature *E. coli *AppA (Gln 23-Leu 432)	-

pFH231	pET23	MH_6_M-mature *E. coli *AppA (Gln 23-Leu 432)	Erv1p

pFH244	pET23	MH_6_M-mature *E. coli *AppA (Gln 23-Leu 432)	DsbC

pFH233	pET23	MH_6_M-mature *E. coli *AppA (Gln 23-Leu 432)	Erv1p + DsbC

Expression from pET23 plasmids is by IPTG induction; all pET23 vectors have a SpeI site added to the multi-cloning site. The co-expressed proteins are either *S. cerevisiae *Erv1p (Met 1-Glu189) and/or the mature form of *E. coli *DsbC (Asp21-Lys236).

The genes for mature *E. coli *alkaline phosphatase (PhoA; Arg22-Lys471), mature *E. coli *phytase (AppA; Gln23-Leu432) and mature DsbC (Asp21-Lys236) were amplified by PCR using a colony of *E. coli *XL1-Blue as a template. The gene for Erv1p (Met1-Glu189) was amplified using a plasmid kindly provided by Prof Thomas Lisowsky as a template.

Erv1p and mature DsbC were cloned into pET23a. An alternative cloning strategy for Erv1p using pET23d (which replaces the 5' NdeI restriction site with NcoI) was also used. This adds an extra Glycine between Met1 and Lys2 of Erv1p. Both versions of the protein were used in a variety of co- and pre-expression experiments and no significant differences between the two were observed. Mature PhoA and AppA were cloned into a modified version of pET23a which includes an N-terminal his-tag in frame with the cloned gene and an additional SpeI site in the multi-cloning site between the EcoRI and SacI sites. The resulting gene products include the sequence MHHHHHHM- prior to the first amino acid of the protein sequence.

Polycistronic vectors were constructed by taking fragments encoding the folding factors from the pET23 based constructs which include the ribosome binding site e.g. XbaI/X fragments and ligating them into the SpeI/X cut plasmid encoding the protein of interest (where X is an appropriate restriction site found in the multi-cloning site after SpeI and not found in either gene e.g. XhoI). After a single such ligation this generates a plasmid that contains a single transcription initiator/terminator and hence makes a single mRNA, but has two ribosome binding sites and makes two proteins by co-expression from two translation initiation sites. This ligation results in the loss of the original SpeI site. Transfer of a SpeI site after the second gene into the new vector allows a third gene to be cloned by the same method resulting in a tricistronic vector which makes three proteins from a single mRNA.

All plasmid purification was performed using the QIAprep spin miniprep kit (Qiagen) and all purification from agarose gels was performed using the Gel extraction kit (Qiagen), both according to the manufacturers' instructions.

All plasmids generated were sequenced to ensure there were no errors in the cloned genes (see Table 3 for plasmid names and details).

### Protein expression

For expression in LB media, *E. coli *strains containing expression vectors were streaked out from glycerol stocks stored at -70°C onto LB agar plates containing suitable antibiotics to allow for selection (100 μg/ml ampicillin for pET23 derivatives, 35 μg/ml chloramphenicol for pLysS derivatives; with 10 μg/ml tetracycline and 15 μg/ml kanamycin for selection of rosetta-gami strains). The next day one colony from these plates were used to inoculate 5 ml of LB media, containing suitable antibiotics (100 μg/ml ampicillin for pET23 derivatives, 35 μg/ml chloramphenicol for pLysS derivatives; with 10 μg/ml tetracycline and 15 μg/ml kanamycin for selection of rosetta-gami strains), and grown overnight at 30°C, 200 rpm. This overnight culture was used to seed a 50 ml culture of LB containing suitable antibiotics in a 250 ml conical flask to an optical density of 0.05 at 600 nm (OD600). The addition of FAD to the media is not required for the production of active Erv1p and preliminary experiments suggestion such addition does not increase the yield of active PhoA or AppA or other proteins tested in this system. This culture was grown at 30°C, 200 rpm until the OD600 reached 0.4 at which point protein production was induced by the addition of 0.5 mM IPTG. The cells were then grown for a total of 4 hours post induction at 30°C, 200 rpm and the final OD600 measured. The cells were collected by centrifugation and resuspended to an OD600 equivalent of 10 (based on the final OD_600 _of the culture) in 20 mM sodium phosphate pH 7.4, 20 μg/ml DNase, 0.1 mg/ml egg white lysozyme and frozen. Such normalization allows for easy correction for differences in the growth rates of the cultures and allows rapid equal total protein loading of samples for activity assay and SDS-PAGE analysis. Cells were lysed by freeze-thawing. Where appropriate, the insoluble fraction was removed by centrifugation and the soluble fraction removed quickly to a new container. Cell lysates or soluble fractions were stored frozen in 1 ml aliquots for further experiments as repeated freeze-thawing clearly influenced the results obtained in a protein dependent manner.

### Protein analysis from lysates

PhoA activity was measured using a continuous assay at pH 8.0 with 4-nitrophenylphosphate as the substrate. Since PhoA folds in the absence of disulfide bonds and is able to gain activity due to air oxidation post lysis all samples containing PhoA included 100 mM NEM in the lysis buffer to block free thiols and to trap the thiol-disulfide status of the protein during analysis. Similarly 100 mM NEM was added to AppA samples. PhoA activity was determined in 96well micro-titer plates at 37°C. Soluble lysate fractions (from OD_600 _normalized cultures) were diluted 100 fold into 20 mM sodium phosphate buffer pH 7.4. 20 μl of this was used in a total assay volume of 200 μl formed by adding 50 μl of the substrate p-nitrophenyl phosphate solution (0.4% w/v in 1 M Tris pH 8.0) to 130 μl of 1 M Tris pH 8.0. The absorbance was recorded at 410 nm every minute up to 20 minutes and the rate of change in absorbance determined by linear-fit. Yields of active protein were calculated relative to the tested system that generated the most active protein (PhoA with Erv1p co-expression in BL21 (DE3) pLysSRARE) based on the rate of change of absorbance determined in the PhoA assay and the final OD_600 _of the culture (see Table 1 for the values of the individual components).

AppA activity was determined in a similar manner to that of PhoA except that the pH optima of the enzyme and absorbance maxima of the chromogenic product meant that a discontinuous assay had to be performed. In place of the PhoA substrate and Tris solutions, 180 μl of p-nitrophenyl phosphate solution (0.4% w/v in 0.25 M Glycine pH 2.5) was used. After 20 min incubation at 37°C the reaction was quenched by the addition of 50 μl of 5M NaOH and the absorbance was measured at 410 nm.

The redox state of PhoA and AppA was determined as follows. 10 ml of culture was spun down and resuspended in 20 mM sodium phosphate pH 7.4, 20 μg/ml DNase, 0.1 mg/ml egg white lysozyme 100 mM NEM to give the equivalent of an OD_600 _of 10 and incubated at 30°C for 5 minutes. 0.5 or 1 ml aliquots were removed for activity measurements (see below) then the samples were frozen at -20C. Cells were then lysed by freeze-thawing. After lysis total protein was precipitated with 10% trichloroacetic acid (TCA; final concentration) on ice for 10 minutes. The pellet was washed with acetone and then resuspended in 2% SDS, 100 mM Tris pH 7.4 with or without 50 mM DTT for 15 min at 37°C. The proteins were precipitated again with 10% TCA, washed twice with acetone and the pellet resuspended in alkylation buffer (2% SDS, 100 mM Tris pH 8.0 containing 5 mM malPEG-2000 (NOF Corporation, Japan)) at 37°C for 1 hour. All samples were reduced with 50 mM DTT before loading onto 12.5% SDS-PAGE and the PhoA and AppA proteins were detected by western blotting using His-tag antibody (Santa Cruz Biotechnology, USA).

### Protein purification

PhoA was expressed as described above and purified by immobilised metal affinity chromatography (IMAC) using TALON superflow metal affinity resin (BD Biosciences, USA) with phosphate present in the lysis and purification buffers. Subsequent analysis (see below) revealed that the purified proteins had no PhoA activity and that all of the purified proteins had a mass 80 Da greater than expected, consistent with phosphorylation. Subsequently, PhoA and AppA were expressed as described above except that the 20 mM sodium phosphate in the lysis buffer was replaced with 50 mM Tris pH 8.0. 100 mM NEM was added to part of the resuspended cell culture, while no NEM was added to the remainder. After cell lysis and removal of the cell debris by centrifugation all samples were purified by IMAC using 400 μl of TALON resin with no phosphate buffer present at any time. Specifically, the resin was washed with 3 mls of water and then equilibrated with 3 mls of equilibration buffer (50 mM Tris, 300 mM sodium chloride, 10 mM immidazole, pH 8.0) before the clarified *E. coli *lysate was loaded. The column was then washed with 5 mls of equilibration buffer before being washed with 3 × 5 mls of wash buffer (50 mM Tris, 300 mM sodium chloride, 20 mM immidazole, pH 8.0), before elution with 2 × 400 μl of elution buffer (50 mM Tris, 300 mM sodium chloride, 250 mM immidazole, pH 8.0). The purified PhoA concentration was determined spectrophotometrically at 280 nm using a calculated molar absorption coefficient (32900 M^-1^cm^-1^). The concentration of purified AppA was too low to determine spectrophotometrically or by Bradford assay, but relative concentrations were estimated by fluorescence measurements performed on a Perkin-Elmer LS50B spectrometer at 25°C, excitation 280 nm, emission 320-400 nm and slit widths 5 nm.

### Analysis of purified proteins

PhoA and AppA activity was measured as per the activity tests on the *E. coli *lysates, but with variable volumes of purified PhoA and 50 μl of purified AppA replacing the lysate. Up to forty-fold more PhoA purified from BL21 (DE3) pLysSRARE was used compared with the other PhoA preparations due to the low specific activity of this protein.

Elman's assay for free thiol content were performed at room temperature under denaturing conditions in 50 mM Tris buffer, 2 M Guanidine hydrochloride (pH 8.0) using 0.073 mg/ml Elman's reagent. The change in absorbance at 412 nm was monitored after 15 minutes and the free thiol content calculated using a molar extinction coefficient of 13600 M^-1^cm^-1^.

Prior to mass spectrometric analysis the protein samples were desalted using pepClean™ C-18 spin columns (Pierce, Rockford, IL, USA) according to manufacturer's instructions. Molecular masses were measured with a Q-Tof-2 electrospray ionisation mass spectrometer (Micromass, UK) using positive ionisation.

## Competing interests

A patent application has been filed.

## Authors' contributions

FH and VDN participated in the design of the research. FH, VDN and KEHS performed the research. LWR conceived and coordinated the study, participated in its design, performed the research and wrote the manuscript. All authors read and approved the final manuscript.
